# The Role of Dysfunctional Adipose Tissue in Pancreatic Cancer: A Molecular Perspective

**DOI:** 10.3390/cancers12071849

**Published:** 2020-07-09

**Authors:** Davide Brocco, Rosalba Florio, Laura De Lellis, Serena Veschi, Antonino Grassadonia, Nicola Tinari, Alessandro Cama

**Affiliations:** 1 Department of Pharmacy, “G. d’Annunzio” University of Chieti-Pescara, Via Dei Vestini 31, 66100 Chieti, Italy; rosalba.florio@unich.it (R.F.); laura.delellis@unich.it (L.D.L.); serena.veschi@unich.it (S.V.); alessandro.cama@unich.it (A.C.); 2 Department of Medical, Oral and Biotechnological Sciences, University “G. d’Annunzio”, Chieti-Pescara, 66100 Chieti, Italy; grassa@unich.it (A.G.); ntinari@unich.it (N.T.); 3 Center for Advanced Studies and Technology (C.A.S.T.), University “G. d’Annunzio”, Chieti-Pescara, 66100 Chieti, Italy

**Keywords:** pancreatic cancer, adipose tissue, obesity

## Abstract

Pancreatic cancer (PC) is a lethal malignancy with rising incidence and limited therapeutic options. Obesity is a well-established risk factor for PC development. Moreover, it negatively affects outcome in PC patients. Excessive fat accumulation in obese, over- and normal-weight individuals induces metabolic and inflammatory changes of adipose tissue microenvironment leading to a dysfunctional adipose “organ”. This may drive the association between abnormal fat accumulation and pancreatic cancer. In this review, we describe several molecular mechanisms that underpin this association at both local and systemic levels. We focus on the role of adipose tissue-derived circulating factors including adipokines, hormones and pro-inflammatory cytokines, as well as on the impact of the local adipose tissue in promoting PC. A discussion on potential therapeutic interventions, interfering with pro-tumorigenic effects of dysfunctional adipose tissue in PC, is included. Considering the raise of global obesity, research efforts to uncover the molecular basis of the relationship between pancreatic cancer and adipose tissue dysfunction may provide novel insights for the prevention of this deadly disease. In addition, these efforts may uncover novel targets for personalized interventional strategies aimed at improving the currently unsatisfactory PC therapeutic options.

## 1. Introduction

Pancreatic cancer (PC) is the fifth most common cause of cancer death in Europe, US and Oceania and it is predicted to become the second most lethal cancer within the next ten years [1,2,3]. Indeed, PC remains one of the most deadly malignancies, with an incidence/mortality ratio of 94% and a 5-year survival rate of only 9% [1,3]. Pancreatic ductal adenocarcinoma (PDAC), which is the focus of this review, accounts for more than 90% of all pancreatic malignancies [4]. Few chemotherapeutic agents and their combinations still represent the unsatisfactory standard of care for the large majority of unresectable patients [5,6].

Adipose tissue (AT) is a type of loose connective tissue and adipocytes represent almost 90% of adipose tissue volume. Different cell types including fibroblasts, endothelial cells, stem and progenitor cells, innate and adoptive immune cells surrounded by a rich milieu of extracellular matrix (ECM) components and signaling molecules participate to the complexity of the adipose tissue microenvironment [7]. The adipose tissue “organ” includes different adipose depots composing white, brown, beige and pink adipose tissue, each involved in systemic and regional control of many physiological processes, including total body energy storage and balance, feeding behavior, hematopoiesis, sexual function and reproduction. This organ contributes to cancer pathogenesis and pathobiology [8,9,10].

Obesity is a well-defined risk factor for several cancer types and is associated with poorer outcomes for some tumor subsets [11,12,13,14]. The complex molecular mechanisms underlying the cancer-obesity link, also defined as “adiponcosis”, are not fully understood [15]. Among factors playing a role in adiponcosis, hypoxia and dysregulation of lipid metabolism, secondary to abnormal fat accumulation, are key promoters of low-grade inflammation of the adipose tissue that leads to dysregulation of its metabolic and secretory functions [16]. However, a similar adipose tissue inflammatory and dysfunctional status may be induced by direct interaction with tumors, independently from obesity [17,18]. A growing number of studies highlighted how altered adipose milieu may drive malignant initiation and progression [19,20]. In this regard, metabolic and inflammatory changes of the adipose tissue mostly occurring in overweight and obese, but also in metabolically obese normal weight (MONW) individuals, deregulate systemic and local physiological homeostasis, thus influencing the course of solid tumor development [10,20]. Altered systemic release of adipokines, growth factors, sexual hormones and cytokines by the whole dysfunctional adipose organ are considered among the main players in cancer development [21]. Moreover, local release of the these factors is likely to be responsible for the increased aggressiveness of tumors invading surrounding adipose tissue [22,23,24,25]. In particular, pro-inflammatory modifications of the adipose tissue microenvironment all create a favorable environment for cancer initiation and progression. These modifications include (1) expansion and recruitment of inflammatory cells, (2) ECM remodeling, (3) stimulation of angiogenesis and lymphoangiogenesis, (4) increased vessel permeability, (5) accumulation of adipose stem/stromal cells (ASCs), (6) local and systemic release of pro-tumor growth factors, cytokines, chemokines, hormones and extracellular vesicles [26,27,28].

Epidemiological studies indicated a solid causal association between obesity and pancreatic cancer, especially for body fatness in childhood, adolescence and young adulthood, which was identified as an emerging risk factor for PC development [29,30,31,32]. Additionally, some studies revealed that high body mass index was associated with increased risk of death for patients affected by PC [33,34,35]. Thus, these epidemiological links suggest a possible role of deranged adipose organ homeostasis in promoting pancreatic cancer initiation and progression.

Efforts to unravel the molecular mechanisms linking dysfunctional adipose tissue and pancreatic tumor may contribute to the discovery of novel targets for improved prevention and treatment of this cancer type. Here we review recent findings about molecular processes underlying the impact of both peripheral and tumor-associated adipose tissue in PC development, as well as interventional strategies targeting adipose organ pathways in PC. MEDLINE database (1950–2020) was searched on March 2020 for literature review. We combined keywords, such as “pancreatic cancer”, “adipose tissue”, “obesity”, “adiposity” and additional keywords identified according to the specific headings discussed in the review. Both basic and clinical research studies were evaluated.

## 2. The Role of Subcutaneous and Visceral Adipose Tissue in Pancreatic Cancer

**Figure 1:**
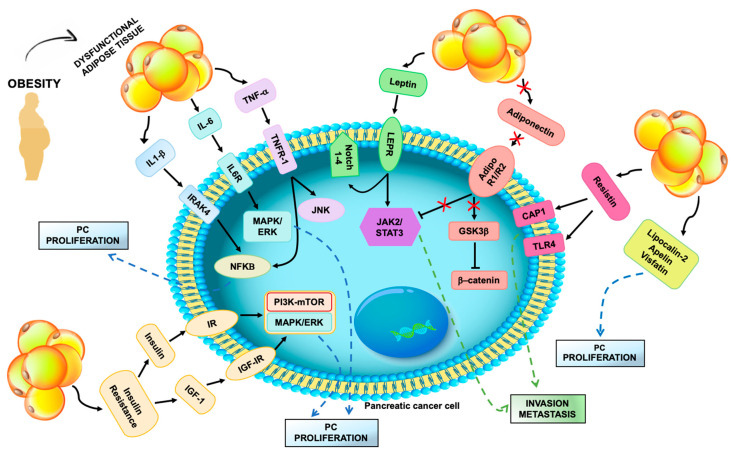
Systemic mediators of dysfunctional adipose tissue effects on pancreatic cancer cell. Excess fat accumulation induces hypoxia and low-grade inflammation in the adipose tissue, resulting in altered secretion of pro-inflammatory cytokines and adipokines. Increased release of TNF-α, Interleukin-1β (IL-1 β) and IL-6 stimulate pancreatic cancer (PC) cell proliferation via activation of the NF-kB, MAPK/ERK and JNK signaling pathways. Elevated circulating leptin may drive pancreatic tumor invasion and metastasis triggering the JAK2/STAT3 axis. Other adipokines, including resistin, lipocalin-2, apelin and visfatin, may also promote PC growth and progression. Reduced release of adiponectin by dysfunctional adipocytes decreases tumor-suppressor effects of adiponectin, mediated by JAK2/STAT3 inhibition and down-regulation of intracellular β-catenin. Expansion and inflammation of visceral adipose tissue induce insulin resistance that fosters systemic secretion of insulin and IGF-1. Activation of insulin receptor (IR) and insulin-like growth factor-I receptor (IGF-IR) enhances PC proliferation through the PI3K/mTOR and MAPK/ERK pathways.

Dysfunctional subcutaneous and visceral adipose tissue can exert a significant role in promoting pancreatic tumor. The first evidences of obesity-mediated pro-tumorigenic activity suggested the importance of altered systemic release of signaling molecules, including insulin, IGF-1, adipokines, cytokines and sexual hormones (Figure 1, Table 1). Recent advances uncovered novel possible mechanisms, such as altered gut microbiome, by which adipose tissue depots outside the pancreatic tumor may influence its growth and progression.

### 2.1. Insulin and Insulin-Like Growth Factor (IGF)

Fat tissue accumulation and obesity are associated with increased risk of type 2 diabetes and insulin resistance development [52]. In particular, high caloric intake followed by expansion and inflammation of white adipose tissue depots are considered responsible for the systemic release of free fatty acids (FFAs). Thereafter, FFAs in excess are stored in non-adipose organs such as liver, muscle, heart, pancreas and kidney, promoting insulin resistance and diabetes onset [53,54]. Several studies demonstrated a role of type 2 diabetes mellitus (T2DM) as a risk factor for PC development [55]. Since hyperinsulinemia and increased bioavailability of insulin-like growth factors (IGFs) are typically associated with insulin resistance in patients with T2DM and obesity, researchers have long considered a pivotal role of insulin/IGF signaling in tumors, including PC [36,56,57]. As a matter of fact, insulin/IGF signaling was found to foster PC cell proliferation and survival through insulin-like growth factor-I receptor (IGF-IR) and insulin receptor (IR)-mediated activation of the PI3K-mTOR and MAPK/ERK signaling pathway, as well as through downregulation of tumor suppressor PTEN [36,37,57,58,59,60].

Besides direct mitogenic effects on pancreatic ductal cells, recent studies highlighted a possible impact of insulin/IGF signaling on stromal cells involved in pancreatic tumor initiation and progression. In particular, hyperinsulinemia and elevated IGF-1 are associated with activation of pancreatic stellate cells (PSCs) that enhance production and remodeling of extracellular matrix [61,62]. Similarly, activation of pancreatic stellate cells (PSC), recruitment of inflammatory cells and ECM remodeling were observed in a PC-prone genetically engineered mouse model of Pdx1-KC (Pdx-1Cre; LSL-KrasG12D) fed high fat calorie-diet (HFCD) [38]. In the same study, mice fed non-HCFD diet presented low serum IGF-1 concentrations, reduced desmoplasia and experienced prolonged survival [38]. In line with these findings, Matsuda et al. reported that Zucker diabetic fatty rats chronically fed high-fat diet (HFD) showed pancreatic acinar cell injury and induction of pancreatic fibrosis [39]. Additionally, IGF-1 was found to directly induce C-C Motif Chemokine Ligand 5 (CCL5) secretion in mesenchymal stem cells neighboring PC cells, promoting malignant cell invasion and migration [63,64]. In turn, activated tumor-associated stromal cells release stromal proteases that foster local free IGF bioavailability by degrading IGF binding proteins [65].

Finally, in PC patients, several clinical studies confirmed the correlation between poorer survival and increased IGF-1 serum concentrations, as well as low blood level of IGF-binding proteins (IGFBPs), supporting the role of IGF-1 signaling in this cancer [66,67,68].

### 2.2. Adipokines

The adipose tissue depots can regulate whole body energy metabolism secreting a variety of soluble factors called adipokines. These molecules, in particular leptin and adiponectin, may be involved in PC development.

#### 2.2.1. Leptin

Leptin is a peptide mainly expressed in white adipose tissue and is involved in regulation of energy expenditure and food intake [69]. Leptin can be released even by non-adipose tissues and modulates other biological function, such as sexual reproduction, angiogenesis, bone remodeling and hematopoiesis [70]. Importantly, increased leptin release and resistance are associated with obesity and a growing body of evidence suggests their involvement in promoting pancreatic cancer progression [71,72,73]. In this context, case-control studies showed that high plasma leptin concentrations were associated with an increased PC risk [74,75].

Leptin exerts its biological activity binding to the leptin receptor, which belongs to class I cytokine receptor family [76]. Hypoxia and hypoxia inducible factor-1 (HIF) were shown to induce the expression of leptin receptor in PC cell lines and were found to be co-expressed in human pancreatic tumor tissue [77]. Furthermore, tumor over-expression of leptin receptor was associated with metastasis and worse overall survival in PC patients [77]. In another study, leptin exerted its pro-tumorigenic activity by promoting migration and invasion of PC cells [40]. PC xenografts over-expressing leptin showed faster growth and higher number of metastatic lymph nodes than controls [40]. In that study, up-regulation of matrix metalloproteinase 13 (MMP13) was detected in both PC cells treated with leptin and in leptin-overexpressing PC xenografts [40]. Furthermore, MMP13 and leptin receptor expression were correlated in metastatic lymph nodes of PC patients [40]. Activation of JAK2/STAT3 signaling in pancreatic cells appeared to be responsible for leptin-induced MMP13 over-expression and, as a consequence, for cancer invasion and metastasis [40]. In another study, leptin was shown to foster cell proliferation through activation of Notch 1–4 receptors and related downstream proteins (Survivin and Hey2) [41]. Inhibition of leptin signaling by leptin peptide receptor antagonist-2 conjugated to nanoparticles (IONP-LPrA2) in MiaPaCa-2 xenografts reduced tumor growth, expression of PC stem markers as well as Notch family receptors and ligands [41].

Lastly, leptin appears to promote chemoresistance of PC cells. In particular, BxPC-3 and MiaPaCa-2 PC cells treated with leptin showed enhanced cell proliferation and production of anti-apoptotic factors, as well as reduced response to 5-Fluorouracil (5-FU) [78]. Moreover, inhibition of leptin effects with IONP-LPrA2 was associated with Notch signaling down-regulation in PC tumorspheres treated with 5-FU [78].

#### 2.2.2. Adiponectin

Adiponectin, or AdipoQ, is secreted by adipose tissue to enhance insulin sensitivity and control whole body energy homeostasis [79]. The physiological activity of adiponectin is mediated by its interaction with two protein receptors, AdipoR1 and AdipoR2, and activation of the 5’ adenosine monophosphate-activated protein kinase (AMPK) pathway [80]. Low serum concentration of adiponectin is associated with increased visceral fat in overweight and obese subjects [81]. Accordingly, a growing body of evidence supports anti-tumor activity of this adipokine [82]. In this regard, a large nested case-control study demonstrated an inverse correlation between blood concentration of adiponectin and PC [83]. In this study, subjects diagnosed with PC presented median plasma adiponectin levels lower than controls, independently from other insulin resistance markers [83]. Accordingly, in Pdx1-KC mice a calorie-restricted diet was accompanied by increased serum adiponectin and by a delayed progression of pancreatic intraepithelial neoplasms to pancreatic ductal adenocarcinoma [42]. Messaggio et al. showed reduced expression of both adiponectin receptors in human and murine pancreatic tumors, as compared to normal pancreatic acinar tissue [84]. In the same study, full-length adiponectin significantly inhibited cell proliferation in PC cell lines [84]. Conversely, AdipoRon, a small molecule agonist for both adiponectin receptors, induced PC cell apoptosis and reduced pancreatic tumor growth, presumably via downregulation of the leptin/STAT3 signaling [84]. A recent study suggested that tumor-suppressor activity of adiponectin in human PC may be also associated to modulation of the β-Catenin pathway, since downregulation of cellular β-catenin levels through the Akt/GSK3-β pathway was found in PC lines treated with adiponectin [43]. Furthermore, the same study showed that knockdown of adipoR1/adipoR2 in human PC xenografts markedly induced tumor growth as compared to controls [43].

#### 2.2.3. Other Adipokines

Lipocalin-2 (LCN2) is a secretory glycoprotein released by adipocytes that is involved in the control of adipose tissue inflammation and in the regulation of peripheral insulin sensitivity [85]. LCN2 is known as a marker of obesity and insulin resistance [85]. Some reports described elevated level of LCN 2 in sera of PC patients [44,86,87]. A genetically modified mouse model of PC employing Lcn2−/−/KRas G12D/Ela-creER mice fed high fat diet (HFD) presented reduced obesity development and extended survival compared with controls. Reduction of inflammation and fibrosis within tumor tissue, as well as decreased PanIN lesion formation were also observed [44]. A possible mechanism by which LCN2 exerts its pro-tumorigenic activity in PC is by modulating PSCs cytokine release, favoring tumor microenvironment (TME) inflammation [44].

A recent study reported that elevated serum levels of resistin, another adipocyte-secreted factor, correlated positively with tumor grade in PC patients [88]. Inhibition of resistin receptors, adenylyl cyclase-associated protein 1 (CAP1) and toll-like receptor 4 (TLR4) in PC cell lines were associated with reduced proliferation, migration and invasion [88].

Other adipocytokines like apelin and visfatin appear to contribute to PC initiation and progression, but their role needs to be further clarified [89,90].

### 2.3. Proinflammatory Cytokines

Healthy adipose tissue is characterized by an anti-inflammatory microenvironment. Specifically, secretion of anti-inflammatory adipokines and infiltration of regulatory immune cells, such as M2 macrophages, Th2 lymphocytes and myeloid-derived suppressor cells, characterize the physiological state of the adipose tissue. Pro-inflammatory transformation associated with pathological expansion of visceral adiposity occur in metabolic disorders like obesity and are matched with a plethora of different secreted adipose-related molecules, as well as with a pattern of adipose stromal immune infiltrate, wherein Th1 cells, neutrophils, natural killer (NK) cells, and monocyte/macrophages are prominent [19]. In particular, pro-inflammatory macrophages, which constitute the typical “crown-like structure” (macrophages surrounding dying or dead adipocytes) of inflamed adipose depots, represent the most abundant cell population in obese dysfunctional adipose tissue and the leading source of pro-inflammatory cytokines as IL-6, TNF-α and IL1-β [91]. Some of the pro-inflammatory factors secreted by infiltrating adipose tissue macrophages (ATMs), other immune cells and adipocytes are elevated in obese subjects and, importantly, they are involved in PC development and progression [92].

#### 2.3.1. TNF-α

One of the main pro-inflammatory cytokine expressed by obese adipose tissue is TNF-α, which was demonstrated to play a significant role in pancreatic tumor promotion and invasion [45,93,94]. In this regard, genetic deletion of tumor necrosis factor receptor 1A (*TNFR1*) in p48-KC mice fed HFD, significantly reduces HFD-induced PanIN formation and pancreatic fibrosis compared to littermate controls without *TNFR1* deletion [45]. Importantly, JNK and NF-kB signaling pathways are triggered by TNF-α. Indeed, constitutive NF-kB activity, which is induced in over two thirds of human PC, enhances survival, invasion, metastasis and treatment resistance of PC cells [95,96,97]. Beyond the direct activity of adipose tissue-derived TNF-α in cancer cells, the pivotal role of this cytokine in supporting adipose tissue inflammation might be even more relevant on pancreatic tumor development and progression. Indeed, TNF-α, via NF-kB activation, controls different steps of the adipose tissue inflammatory process, including: chemokine release by adipocyte and pre-adipocytes; subsequent pro-inflammatory M1 macrophage recruitment; macrophage local and systemic secretion of other pro-inflammatory cytokines, such as IL-6 and IL-1beta, which are involved in PC promotion [20,92,98,99].

#### 2.3.2. IL-6

Due to its elevated production in dysfunctional adipose tissue and to its pro-tumorigenic activity in PC, IL-6 is considered another key effector of inflammation-associated tumorigenesis in this cancer subtype [100,101,102]. IL-6 promotes PC proliferation and invasion by inducing activation of STAT3 and JAK2 canonical signaling pathways, which are crucial for development and progression of K-RAS mutated PC in mouse [103,104]. A similar role was also supported by analysis of IL-6/STAT3 signaling in human PC [104]. Recent studies show that invasive properties of PC are also enhanced by IL-6-mediated activation of the small GTPase cell division cycle 42 (CDC42) through a non-canonical transduction of IL-6 signaling [105]. Furthermore, in a iKRAS (p48-Cre;R26-rtTa-IRES-EGFP;TetO-KrasG12D) mouse model of pancreatic cancer, IL-6 appeared to be required for pancreatitis-driven PanINs formation [46]. IKRAS mice presented increased progression to high-grade PanINs, higher tumor cell proliferation rate and prolonged survival, compared to mice with IL-6 deficiency. Moreover, IL-6 pro-tumorigenic function was modulated by the MAPK/ERK signaling pathway [46].

#### 2.3.3. IL-1

Other important pro-inflammatory molecules in the pathogenesis of PC belong to the IL-1 family [106]. Circulating and local expression of IL-1α and IL-1β are positively correlated with adipose tissue inflammation and obesity [107]. Moreover, higher PC risk was observed in subjects with IL1β gene promoter single nucleotide polymorphisms (SNPs) associated with an increased IL-1β secretory phenotype [108]. Accordingly, recent studies show that IL-1β contributes to PC pathogenesis by IL-1 receptor-associated kinase 4 (IRAK4)-mediated activation of NFkB and by triggering the JAK2/STAT signaling in TME, particularly in cancer-associated fibroblasts (CAFs) [109,110].

Overall, pro-inflammatory cytokines promote pancreatic tumorigenesis both directly in cancer cells and, indirectly, by regulating release of other cytokines, supporting adipose tissue pro-inflammatory state and modulating tumor-stroma interactions [99]. Activation of common transcriptional factors involved in the inflammatory response and in PC development, such as NF-kB or STAT3, characterizes the pro-tumorigenic activity of cytokines released by dysfunctional adipose tissue.

### 2.4. Sexual Hormones

The ability of adipose tissue to store and metabolize sex steroids is well known [111]. Fat accumulation and obesity or menopause transition can alter adipose sex hormones metabolism and, thus, their systemic release [112,113]. In particular, hypertrophy of adipose tissue increases adipocyte-mediated conversion of androgens, including androstenedione, into estrogens. The influence of sex hormones in cancer promotion and progression has been largely explored and highly estrogen-responsive tumors, such as endometrial or breast cancer, show among the highest relative risk increase between normal-weight and obese subjects [114,115]. Both biosynthetic enzymes and receptors for these hormones were found to be expressed in normal pancreatic tissue and cancer, supporting their sensitivity to sex steroids [116,117]. Nevertheless, the real impact of sex hormones in PC is not clear. Most epidemiological studies and animal models support a protective role of estrogens, which is at odds with their pro-tumorigenic role in other cancers [118,119,120,121,122]. In this regard, epidemiological studies showed that women present a lower PC risk [117]. No reproductive factor, including age at menarche, parity, breastfeeding, and age at menopause have been associated with PC development, while estrogen-only hormone therapy was associated with an almost 40% reduction of PC risk [118,119]. A protective effect of estrogen in PC is also suggested by mouse models [120,121,122]. Moreover, findings from another study showed a strong gender difference in PC promotion between p48-KC mice fed HFD, with male mice presenting earlier development and significantly higher rates of PC, as compared to female counterpart [123]. The reason for this gender imbalance is not clear but it has been proposed that the lower risk of PC in female mice might be related to the protective effects of estrogens that reduce pro-tumorigenic visceral adipose tissue accumulation [123]. Therefore, this view underlines a link between adiposity, estrogen and PC tumorigenesis.

### 2.5. Gut Microbiota

**Figure 2:**
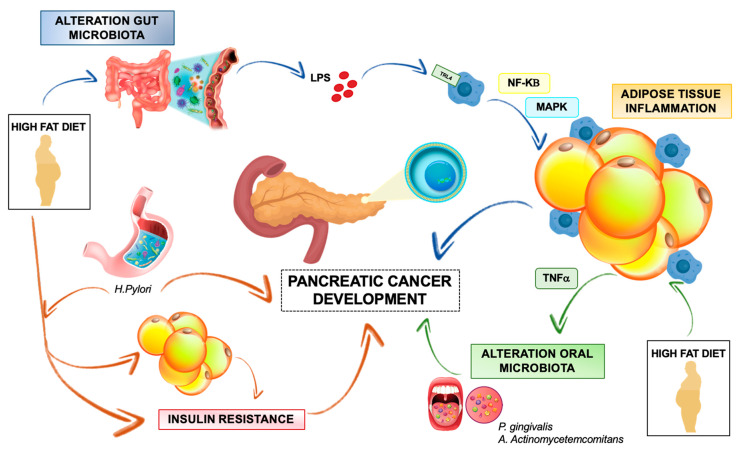
Overview of interactions between gut microbiota, adipose tissue and pancreatic cancer development. High fat diet (HFD)-induced gut dysbiosis promotes systemic lipopolysaccharide (LPS) release and subsequent activation of TRL4 receptor in adipose tissue macrophages. Resulting adipose tissue inflammation contributes to PC development (blue lines). HFD-promoted adipose tissue inflammation favors periodontal inflammation and oral colonization by pathogen bacteria that are related with increased risk of PC (green lines). Gastric colonization by *Helicobacter pylori* fosters HFD-mediated central obesity and insulin resistance, both of which promote PC growth. PC may also be favored by direct translocation of *Helicobacter pylori*-derived protein antigen toward pancreatic tissue and subsequent pancreatic inflammation (orange lines).

The gut microbiome is implicated in several human diseases including cancer, obesity and other metabolic disorders [124,125]. Alterations of gut microbiome, typically associated with HFD and obesity, lead to the activation of a systemic low-grade inflammation response, which is a key shared trigger for adipose tissue dysregulation, insulin resistance and cancer development [126]. Recent studies revealed that perturbations of gut microbiota are directly linked with the onset and progression of gastrointestinal tumors, including PC, by regulating inflammation and immune function (Figure 2) [127]. In particular, PC is associated with a low gut microbiota diversity and a unique gut microbiome profile, characterized by an increase of various pathogens and lipopolysaccharides (LPSs)-producing bacteria, together with a reduction of butyrate-producing bacteria [128]. These features resemble to the microbiome alterations and related host metabolic implications observed in diet-induced obesity and metabolic inflammation [129]. In detail, HFD-induced dysbiosis may affect some properties of gut barrier physiology, including tight junction proteins (TJP) distribution, mucous layer integrity, as well as intestinal immune cell abundance and function, leading to increased gut permeability and release of microbiota-derived products, like LPS, into circulation [130]. Subsequently, LPS binding to Toll-like receptors (TLRs) and to CD14 co-receptors on myeloid cells promotes activation of the myeloid differentiation primary response protein (MYD88) and subsequent activation of MAPK and NFkB-dependent signaling pathways. This fosters a low-grade inflammatory response in several host targets such as adipose tissue, liver, muscle and, more importantly, within tumors [126,131,132]. In this context, it was shown that LPS accelerates pancreatic carcinogenesis by TLR4 activation and induces a fibro-inflammatory response [133]. Furthermore, Sethi et al. demonstrated that gut microbiome modulation using broad-spectrum antibiotic therapy in a pancreatic cancer mouse model may slow tumor growth and increase infiltration of anti-tumor mature T cells [132]. Similarly, an antigenic peptide of *Helicobacter pylori,* associated with autoimmune pancreatitis and PC pathogenesis, may directly translocate from the gut to pancreatic tissue and promote local NFkB-dependent expression of pro-inflammatory cytokines involved in PC progression [134]. Moreover, *Helicobacter pylori* may aggravate HFD-induced systemic inflammation, insulin resistance and central obesity accumulation, thus contributing to PC development (Figure 2) [135].

Overall, gut microbiome may contribute to PC development by triggering and amplifying inflammation response both in the adipose tissue and within pancreatic TME. Recent evidences of increased risk of periodontitis and altered oral cavity microbiota in obese subject suggest the hypothesis that high adipose-related secretion of TNF-alpha may promote periodontal inflammation and infection, highlighting a possible role of pro-inflammatory cytokines released by adipose tissue in the pathogenesis of periodontal disease [136]. Considering that detection of oral pathogens, like *Porphyromonas gingivalis and Aggregatibacter actinomycetemcomitans,* is associated with increased risk of human PC, it is conceivable that inflamed adipose tissue may indirectly contribute to PC development by modulating gut microbiota composition and functions [126,137].

## 3. The Role of Local Adipose Tissue in Pancreatic Cancer

The interaction between PC and local adipose tissue, in the form of pancreatic intravisceral or perivisceral depots, is gaining increasing interest for its possible implications in PC pathogenesis. Indeed, adipocytes as well as stromal adipose cells can interact locally with pancreatic tumor cells and modulate TME, thus triggering most of PC hallmarks [138]. In this section, we review the possible mechanisms regulating this complex interplay, focusing on the pro-inflammatory modification of TME induced by the adipose tissue and on the crosstalk between adipocyte and tumor cell.

### 3.1. Adipose Tissue Inflammation and Pancreatic Cancer

PC is typically characterized by a remarkable fibro-inflammatory reaction. Activation of PSCs, extracellular matrix accumulation, infiltration of myofibroblast-like cells and abnormal vascular supply characterize PC-related desmoplasia, promote growth, progression and PC treatment-resistance [139]. As previously described, adipocyte dysfunction and immune cell recruitment in white adipose tissue occurring in pathological conditions like obesity are responsible of cytokine production, inflammation and fibrosis [20]. Fat accumulation (steatosis) in normal pancreas is a well-known clinical-pathological entity, typically associated with obesity, diabetes and metabolic syndrome, and may trigger a pro-tumorigenic inflammation reaction within pancreas leading to abnormal local release of cytokines, remodeling of ECM components and fibrosis [19,39,140]. As a matter of fact, human pancreatic tumors are characterized by an increased adipocyte content and in more than 20% of PC patients adipocytes infiltrate the normal pancreatic tissue [141]. Moreover, pancreatic steatosis and infiltration of the peri-pancreatic fat (PPF) is associated with poor clinical outcomes in PC patients [142,143].

In this respect, Inicio et al. demonstrated how obesity-associated adipocyte accumulation promotes tumor progression by supporting a pro-inflammatory and pro-fibrotic tumor milieu [47]. In that study, pancreatic tumors developed in KPC (Ptf1-Cre/KrasLSL-G12D/+/Trp53LSL-R172H/+) and iKRAS (p48-Cre; R26-rtTa-IRES-EGFP; TetO-KrasG12D) mice fed HFD were investigated. A significant increase in adipocyte number and size was found in the TME of obese mice. Additionally, an abundant desmoplastic reaction in adipocyte-enriched areas, as well as alongside the tumor infiltration front of adjacent visceral adiposity, was reported [47]. Increased desmoplasia was also associated with impaired vascular perfusion and decreased treatment response [47]. Importantly, release of IL-1β by hypoxic dysfunctional adipocytes was suggested to induce recruitment of tumor-associated neutrophils (TANs), T-regs lymphocytes and activation of PSCs, thus leading to an immunosuppressive TME, ECM remodeling, and PC progression in obese mice [47]. Similar findings were observed in human obese PC patients [47]. Accordingly, inflammation and extensive stromal collagen deposition were shown to appear already during first steps of pancreatic tumor development in mice harboring oncogenic Kras mutations and fed HFD [144]. This supports the possibility that activity of oncogenic Kras and its downstream pathways, including COX2 and phospho-ERK, may be fostered by pancreatic inflammation in mice fed HFD [144]. Consequently, increased COX-2 expression, infiltration of macrophages and activation of PSCs, resulting from increased Kras activity, further promote fibro-inflammation, establishing a feed-forward loop [144]. In a Pdx1-KC mouse model of pancreatic cancer, with mice fed HFD, significant inflammatory immune cell infiltration and elevated pro-inflammatory cytokines expression were detected in the pancreatic tissue and correlated with PanIN lesion development [38]. Robust inflammation and a specific cytokine profile were also observed in the peri-pancreatic fat (PPF) of wild-type and KC mice fed HFD. This may further contribute to inflammation and tumor progression in the adjacent pancreatic tissue [48]. Interestingly, PPF appears histologically and functionally different from adipose tissues surrounding other visceral organs and there is evidence suggesting its involvement in the development and progression of PC [48,143,145].

Overall, cumulating evidence outlines a model in which activation of an inflammatory response in the intra- or peri-pancreatic adipose tissue may in turn induce inflammation and fibrosis in the neighboring pancreatic tissue, thus promoting PC progression and treatment resistance. Cytokines and other soluble factors released by adipocytes and by adipose-infiltrating immune cells represent possible mediators of this process.

### 3.2. Cancer-Associated Adipocytes and Adipose Stromal Cells in Pancreatic Cancer

**Figure 3:**
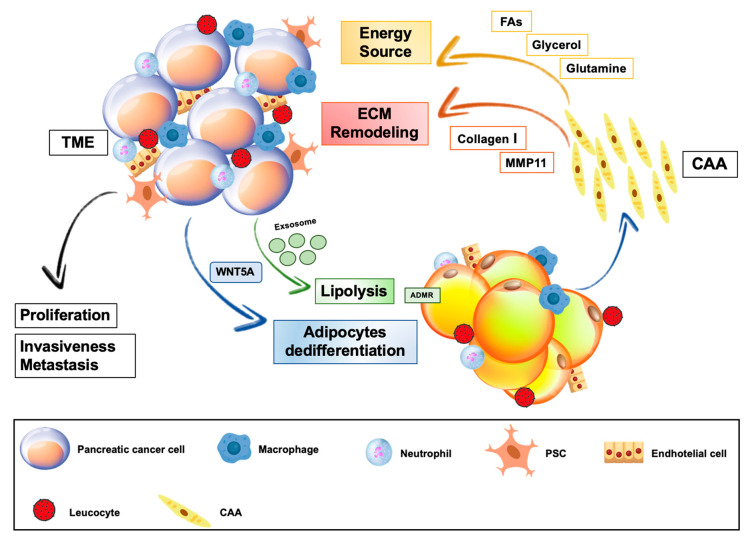
Crosstalk between local adipose tissue and pancreatic cancer. Pancreatic tumor cells stimulate adipocyte dedifferentiation and lipolysis, thus promoting transformation of local adipocytes into cancer associated adipocytes (CAAs). Delipidation and impaired metabolic homeostasis of CAAs are associated with increased release of fatty acids (FAs), glycerol and glutamine, which are used as energy source for cancer cell growth. Increased expression of MMP11 and collagen I, secondary to acquired fibroblast-like properties of CAAs, promotes extracellular matrix (ECM) remodeling within tumor microenvironment (TME), favoring PC invasiveness and metastasis.

The identity of cancer-associated adipocytes (CAAs) has been well recognized in some type of cancer, such as breast cancer [146,147]. Morphological, functional and epigenetic changes of tumor-surrounding adipocytes can be driven by the crosstalk with malignant cells, which gives rise to a unique tumor-associated cell type [148,149,150]. As described above, there are evidences that the presence of intra-pancreatic and peritumoral adipocytes confers greater aggressiveness to PC [47,142,143]. Recent studies uncovered novel mechanisms by which pancreatic cancer cells induce a phenotypic remodeling of neighboring adipocytes and establish a favorable interaction with adipose cells (Figure 3). 

In this regard, co-culture of mature adipocytes with Panc-1 and Mia PaCa2 cell determined delipidation, impaired lipid homeostasis and dedifferentiation as the most significant elements for the process of transformation of mature normal adipocytes into CAAs [49]. Indeed, CAAs act as an energy source for tumor cells and contribute to tumor growth by releasing fatty acids (FAs) and glycerol, which represent the main lipid precursor employed for energy purposes by malignant cells (Figure 3) [148]. Hypoxic TME induce acceleration of lipolysis and release of FAs for cancer cells, thus reducing the lipid content of adipocytes [148,149]. Additionally, PC cells may directly stimulate adipocyte lipolysis [25]. Actually, co-culture of pancreatic cancer cells with mature adipocytes caused a reduction of adipose cell size in parallel with an increase of hormone sensitive lipase (HSL) expression and FAs concentration in the culture medium [25]. Moreover, enhanced FA uptake into PC cells, after incubation with adipose-tissue derived conditioned medium, was associated with increased tumor cell migration and invasiveness [25]. Accordingly, signs of cancer-induced lipolysis were observed in the adipose tissue adjacent to the invasive front of human PC [25]. Possible mediators of lipid metabolism reprograming in CAAs may be extracellular vesicles. In this regard, it was shown that PC is able to directly stimulate lipolysis through the exosome-mediated activation of the adrenomedullin (AM)/andromedullin receptor (ADMR)/ERK pathway in adipocytes [150].

Other nutrients, such as amino acids, may be transferred from adipocytes to tumor cells strengthening the metabolic symbiosis. PanIN and invasive PC are glutamine-dependent for growth [151]. In co-culture of mature adipocytes and PC cells, adipocyte-mediated stimulation of tumor cell proliferation was amplified in nutrient-poor media, characterized by the absence of glutamine [151]. Irreversible inhibition of adipocyte glutamine synthetase and of cellular glutamine transport disrupts adipocyte-induced PC cell growth [151]. Moreover, PC cells down-regulate the expression of adipocyte glutaminase, thus promoting glutamine secretion [151]. Besides metabolic reprogramming, tumor-associated adipocytes may undergo a mesenchymal dedifferentiation process. In this regard, dedifferentiation of mature adipocytes toward a fibroblast-like phenotype was observed *in vitro*, after co-culture of 3T3-L1 adipocytes and MiaPaCa2 [50]. RT-PCR revealed loss of mature adipocyte gene profile and increased expression of fibroblast-specific genes, including MMP11, collagen I and α-SMA, in dedifferentiated adipocytes [50]. In the same study, it was suggested that WNT5a signaling has a crucial role in adipocytes dedifferentiation. This role of WNT5a signaling was supported by the observations that the pathway was up-regulated in co-cultured adipocytes, which undergo dedifferentiation, while selective inhibition of WNT5a was able to block dedifferentiation of adipocytes into fibroblast-like cells. [50]. It was proposed that IL-6, released by adipocytes in pro-inflammatory condition, promotes WNT5a expression in PC cell and this in turn mediates phenotypic remodeling of tumor-associated adipocytes [50]. Similar findings were recently reported by another in vitro study, in which morphological and epigenetic signs of mesenchymal differentiation were described in adipocytes co-cultured with Panc-1 and MiaPaCa2 tumor cells [49]. Of note, whole transcriptome analysis of co-cultured adipocytes showed that transcripts modulated by this co-culture were significantly enriched in pathways linked to remodeling of pancreatic TME, such as “cytokine-mediated signaling pathway”, “angiogenesis” and “ECM organization” [49]. According to these findings and observations from studies exploring the role of CAAs in other cancer types, it will be important to investigate whether even in PC, tumor cells, HFD or other factors induce adipocyte dysregulation, fostering local secretion of adipokines, growth factors, pro-inflammatory cytokines or chemokines [19,20,26,47,146,152]. This paracrine activity of adipocytes may target both cancer and stromal cells, promoting PC progression [19,47].

Adipose tissue-derived stromal cells (ASCs) are distributed around capillary vessels in normal adipose tissue and are characterized by pluripotent differentiation capacity toward several lineages of mesenchymal cells [153]. Tumors recruit ASCs from the white adipose tissue by a chemokine gradient and, once reached the TME, ASCs are able to promote several cancer hallmarks, in particular neo-angiogenesis [154,155]. Moreover, obesity drives expansion of white adipose tissue, increased systemic circulation and tumor infiltration of ASCs, which finally may differentiate into pericytes and, then, adipocytes within the TME [156]. ASCs were first reported to stimulate migration and proliferation in pancreatic cancer cell lines SW1990 and PANC-1 via the SDF-CXCR4 axis [157]. The impact of ASC in PC progression was investigated in a recent study combining in vitro and in vivo experiments [158]. This study confirmed that PC cells were able to recruit ASCs. Actually, PC tumors inoculated in visceral fat were characterized by increased growth and ASCs infiltration compared to those inoculated into the pancreas [158]. When ASCs were cultured in cancer cell conditioned medium, they produced dense collagen matrices and fostered migration of cancer cells [158]. The authors suggested that ASCs might promote tumor progression and extra-pancreatic invasion also by rearranging matrix structures [158]. Inhibition of ASC recruitment or activation could potentially lead to novel treatment strategies for PC.

## 4. Targeting Adipose Tissue in Pancreatic Cancer

Based on the growing evidence for a substantial involvement of the adipose tissue in development and progression of some tumors, including PC, it is conceivable that targeting dysfunctional adipose cells could represent an effective strategy to treat cancer [20]. 

Considering the link between PC development and HFD- or obesity-induced adipose tissue inflammation, interventional approaches aimed at targeting inflammation within adipose tissue as well as its promoting effects might constitute an active field of research (Table 2). Low-fat diet and physical activity are associated with significant reduction of white adipose tissue low-grade inflammation, IL-6 release and macrophage infiltration, as well as decrease of pancreatic cancer risk [159,160,161,162]. In this regard, two ongoing randomized trials are investigating the effect of nutritional and/or exercise treatment in patients with early or advanced PC (NCT03187951) [163]. Selective inhibition of major pro-inflammatory cytokines secreted by dysfunctional adipose tissue has been considered for PC treatment, because they act as mediators of PC development and progression. Although pre-clinical evidence demonstrated reduction of pancreatic tumor-associated inflammation and desmoplasia through TNF-α inhibition, randomized clinical studies showed that modulation of TNF-alpha activity in PC patients did not produce any clinical benefit [164,165,166]. Studies in PC cell lines and orthotopic xenografts revealed that targeting IL-1 signaling is a promising strategy and two phase 1 studies evaluated safety of the combination of IL-1 receptor inhibitor Anakinra in association with chemotherapy in advanced PC patients (NCT02021422) (NCT02550327) [167,168]. Another key pro-inflammatory cytokine is IL-6 and the anti-tumor impact of its inhibition has been extensively studied in vivo in combination with both chemotherapy and immunotherapy with an anti-PD1 agent [169,170,171]. Toclizumab, an anti-IL-6 receptor antibody, combined with chemotherapy has already been investigated in a small phase 1/2 trial, while another phase 2 randomized study, comparing toclizumab plus nab-paclitaxel and gemcitabine to chemotherapy alone in locally advanced or metastatic PC patients, is still recruiting (NCT02767557) [172].

Pharmacological interventions might be also focused on targeting insulin resistance and the insulin/IGF signaling, which are considered pivotal link factors between dysfunctional adipose tissue and PC [173]. In this context, one of the most extensively studied agents is metformin. Of note, an in vivo mouse model of PC mimic obesity/T2DM demonstrated that metformin reduced desmoplasia in pancreatic tumor by directly inhibiting the AT1/TGF-β/STAT3 signaling and reducing release of collagen-I/HA by PSCs [174]. Considering that activation of PSCs represents a target of pancreatic dysfunctional adipocytes in obesity and is associated with reduced response to conventional treatment, metformin should be considered as an interesting investigational agent for personalized treatment in obese PC patients [47]. In this regard, metformin was shown to reduce risk of PC [175].

However, despite several lines of pre-clinical and clinical evidence indicating an anti-tumor activity of metformin in PC, randomized clinical trials adding this drug to standard chemotherapy failed to show advantages in clinical outcomes [176,177,178,179,180,181]. Another possible strategy to target the insulin/IGF signaling in PC is represented by the inhibition of IGF-1R and findings from in vivo studies suggested the importance of IGF-1R/ErbB3 combined inhibition to overcome therapeutic resistance to IGF-1R blockade [182]. However, IGF-1R inhibitors, cixutumumab and ganitumab did not improve the efficacy of chemotherapy in randomized trials enrolling metastatic PC patients [183,184].

Lipid metabolism reprogramming is crucial for cancer cell survival under metabolic stress and it is promoted by interaction between tumor cells and surrounding adipocytes [185]. Therefore, disruption of tumor and stromal lipid metabolism may lead to reduced PC proliferation and survival [186]. Several molecules have been developed and investigated in pre-clinical and clinical studies. Among FA synthesis inhibitors, BAY ACC022 and SB-204990, respectively targeting the Acetyl-CoA carboxylase (ACC) and ATP citrate lyase (ACLY), reduced tumor growth in pancreatic cancer xenograft models [187,188]. In this context, promising findings in PC pre-clinical studies have been also obtained by blocking different domains of the multi-enzyme complex fatty acid synthase (FASN) with the two agents, epigallocatechin-3 gallate and orlistat [189,190]. In addition, inhibition of *de novo* cholesterol synthesis has been considered for PC treatment. Statins have been associated with decreased human PC risk and reduction of PC development and growth in pre-clinical studies conducted in vivo and in vitro [191,192,193]. Despite retrospective findings suggest increased survival in PC patient treated with statins, a phase 2 randomized study, evaluating efficacy and safety of gemcitabine plus simvastatin, as compared to gemcitabine plus placebo in advanced PC patients, did not show improvement of patient clinical outcomes in the investigational arm [194,195]. A phase 3 clinical trial investigating efficacy of atorvastatin in PC is underway (NCT02201381). Interestingly, targeting lipid metabolic reprograming may represent a strategy to overcome resistance to other anti-cancer drugs, such as antiangiogenic agents [196]. In fact, increased resistance to antiangiogenic therapy characterizes tumors with higher adipose infiltration. Indeed, antiangiogenic-induced hypoxia pushes a metabolic shift from glycolysis to fatty acid oxidation (FAO) in tumor-associated adipocytes and, subsequently, promotes increased cancer cell proliferation and FA uptake [196]. Combination of anti-VEGF treatment with a FAO inhibitor restores antiangiogenic agent sensitivity in cancer cells growing in an adipose environment and may represent a strategy to be investigated in PC [196].

Sensitivity of PC cells to glutamine metabolism abrogation has been also explored. In this context, a modest anti-tumor activity of glutaminase inhibitors (GIs), BPTES and CB-839, was demonstrated in PC pre-clinical studies [197,198]. Of note, improvement of drug delivery strategies with GI nanoparticles encapsulation and combination with other drugs able to overcome metabolic resistance to these agents were associated with significant improvement in growth control of PC patient-derived orthografts [198].

## 5. Conclusions

Growing evidence shows that dysfunctional adipose tissue, typically associated with obesity, is closely related to pancreatic cancer promotion and progression. Low-grade inflammation of adipose tissue is responsible of aberrant systemic release of adipokines, inflammatory cytokines, chemokines, hormones and growth factors, which enhance PC development by directly promoting cancer transformation, as well as establishing a favorable environment for tumor growth. Moreover, fibro-inflammatory modification of tumor stroma and pro-tumorigenic metabolic, as well as phenotypic alterations of tumor-associated adipocytes are promoted by the interaction between PC and local adipose milieu. As a result, altered local and peripheral adipose tissue may influence the course of pancreatic tumor progression and its treatment resistance.

Even though recent research advancement led to a broader knowledge of the links between adiposity and PC, many questions remain to be answered. In particular, more efforts are needed to identify the molecular mechanisms regulating the interplay between adipose and cancer cells in the context of TME. Actually, the nature and the role of adipose tissue-derived soluble factors, as well as other possible signaling mediators, such as extracellular vesicles, participating in the crosstalk between adipose tissue and PC cells, need to be further explored. Furthermore, few studies focused on the non-adipocyte cell populations of adipose tissue neighboring PC. Specifically, the recruitment mechanisms of non-adipocyte cells and the individual contribution of different cell lineages within adipose tissue compartment to PC progression need to be investigated. This becomes especially important when considering adipose tissue microenvironment modifications in the context of obesity. Finally, a better understanding of the molecular mechanisms regulating the interaction between adipose tissue and PC may provide novel treatment targets for the prevention and treatment of this lethal disease. However, giving a wider evidence concerning the impact of obese adipose tissue in pancreatic tumor biology and the rising global incidence of obesity, future interventional strategies targeting dysfunctional adipose tissue should be personalized, focusing on identifying differences in outcomes between obese and lean patients in response to specific treatments.

## Figures and Tables

**Table 1:** Summary of main in vitro and in vivo studies investigating the link between dysfunctional adipose tissue and pancreatic cancer.

Study Design	Experimental Model *	Study Aim	Main Results	Ref.
*In vitro*	ASPC-1 and COLO-357 human pancreatic cancer cells	To assess the role of IGF-1 and IGF-1R in human pancreatic cancer	IGF-1 promotes PC cell growth; selective inhibition of IGF-1R reduces cancer cell growth	[36]
*In vivo*	LSL-Kras^G12D^/Pdx-1-Cre/Ink4a/Arf^lox/+^ mouse model	To assess effect of dietary energy balance modulation on pancreatic cancer development and progression through an insulin-like growth factor (IGF)-I-dependent mechanism	Calorie restriction diet (CRD) reduces serum IGF-I, tumoral Akt/mTOR signaling, pancreatic desmoplasia and progression to pancreatic ductal adenocarcinoma (PDAC); CRD increases pancreatic tumor-free survival.	[37]
*In vivo*	KC (Pdx-1Cre;LSL-Kras^G12D^) mice	To develop a model of diet-induced obesity and pancreatic cancer development	Diet high in fats and calories promotes early pancreatic neoplasia, pancreatic TME inflammation, serum IGF-1 elevation and PSCs activation	[38]
*In vivo*	Zucker diabetic fatty (ZDF) rats	To study biochemical and histological changes in the pancreatic exocrine tissue of obese model rat	Chronic HFD is associated to fat accumulation in pancreatic acinar cells, pancreatic fibrosis and acinar cell injury	[39]
*In vivo*	Subcutaneous and pancreatic injection of PANC-1 cells in athymic BALB/c nude mice	To investigate the impact of leptin on pancreatic cancer growth and metastasis	Leptin overexpression in pancreatic tumors is associated with tumor growth, lymph node metastasis and increased MMP-13 expression	[40]
*In vitro/* *In vivo*	BxPC-3, MiaPaCa-2, Panc-1, AsPC-1 cell lines and tumorspheres; Subcutaneous injection of MiaPaCa-2 cells in nude CD1 nu/nu male mice	To assess the role of leptin and Notch pathway in pancreatic cancer progression	Leptin induces cell proliferation and expression of Notch receptors, ligands and downstream molecules; treatment with IONP-LPrA2 decreases tumor growth.	[41]
*In vivo*	KC (Pdx-1Cre;LSL-Kras^G12D^) mice	To investigate the effect of calorie restriction in preventing pancreatic intraepithelial neoplasms (PanINs) development and in delaying progression to pancreatic ductal adenocarcinoma (PDAC)	Intermittent and chronic calorie restricted diets are related with reduced incidence of PanINs, delayed PDAC progression, increased serum adiponectin and decreased serum leptin.	[42]
*In vitro/* *In vivo*	BxPC-3 and CFPAC-1 cell lines; Subcutaneous injection of BxPC-3 cells in athymic BALB/c nude mice	To investigate the effect of adiponectin on human pancreatic cancer	Adiponectin inhibits PC cell proliferation, blocks GSK-3β expression and intracellular accumulation of β-catenin	[43]
*In vivo*	KRas^G12D^/Ela-creER(CRE) KRas/CRE mice bred with Lcn2^−/−^ mice (Lcn2^−/−^/KRas/CRE); C57BL/6 strain mice injected with cells derived from a pancreatic tumor of a KPC mouse model	To study the effects of Lcn2 depletion on obesity, inflammation and PDAC development	Depletion of Lcn2 reduces extracellular matrix deposition, immune cell infiltration, PanIN formation and tumor growth; Lcn2 knockout increases survival in both mouse models.	[44]
*In vivo*	LSL-Kras^G12D^ mice crossed to p48-Cre and Ela-CreER animals; p48-KC mice further crossed to TNFR1-deficient animals	To investigate a possible link between HFD-induced obesity and PanIN development	HFD promotes PanIN development; TNFR-1 depletion reduces HFD-enhanced PanIN development.	[45]
*In vivo*	iKRAS (p48-Cre;R26-rtTa-IRES-EGFP;TetO-Kras^G12D^) crossed with IL-6 deficient mice	To investigate the role of IL-6 in maintenance and progression of pancreatic cancer precursor lesions	Depletion of IL-6 abrogates pancreatic cancer progression	[46]
*In vivo*	KPC (Ptf1-Cre/Kras^LSL−G12D/+/^Trp53^LSL−R172H/+^) and iKRAS (p48-Cre;R26-rtTa-IRES-EGFP;TetO-Kras^G12D^) mice	To investigate the mechanisms of obesity-induced pancreatic cancer progression and treatment resistance	Obesity promotes desmoplasia, tumor growth and impaired efficacy of chemotherapeutics; Inactivation of PSCs, IL1β inhibition, or TAN depletion reduce obesity-induced tumor growth.	[47]
*In vivo*	KC (Pdx-1Cre;LSL-Kras^G12D^) mice	To study the impact of high fat, high calorie diet (HFCD) on visceral adipose inflammation	HFCD enhances inflammation in the visceral adipose tissue (VAT), particularly in peri-pancreatic fat (PPF), and in pancreatic tissue.	[48]
*In vitro*	Co-culture of 3T3-L1 mature adipocytes with Panc-1 and Mia PaCa2 cell lines	To investigate the interaction between mature adipocytes and pancreatic cancer cells	Pancreatic cancer cells induce mature adipocyte delipidation, lipid homeostasis dysregulation and dedifferentiation	[49]
*In vitro*	Co-culture of 3T3-L1 mature adipocytes with Mia PaCa2 cell lines	To study the crosstalk between adipocytes and pancreatic cancer cells	Co-culture induces WNT5a-mediated mature 3T3-L1 adipocytes dedifferentiation to fibroblast-like cells.	[50]

* Reported in vivo models of PC were extensively reviewed by DeCant et al. [51].

**Table 2:** Overview of ongoing and completed clinical trials investigating potential targets involved in the crosstalk between adipose tissue and PC.

Inhibitor Name	Main Molecular Target	Stage of Clinical Trial	Register Trial Code	Status/Results	Notes
Etanercept	TNF-α	Phase I/II	NCT00201838	Completed. Safe; Not improved TTP and OS	Etanercept +/− Gemcitabine in advanced PC
Anakinra	IL-1R	Phase I	NCT02021422	Completed. Safe.	Anakinra + FOLFOX in metastatic PC
Anakinra	IL-1R	Phase I	NCT02550327	Completed. No results available.	Anakinra + cisplatin + nab−paclitaxel + gemcitabine in resectable or potentially resectable PC
Tocilizumab	IL-6R	Phase I/II	JapicCTI-090889	Completed. Tolerable; Inconclusive results.	Tocilizumab + gemcitabine in advanced PC
Tocilizumab	IL-6R	Phase II	NCT02767557	Recruiting	Tocilizumab+/−gemcitabine + nab−paclitaxel in unresectable PC
Metformin	mTOR-ATM/LKB1/AMPK	Phase II	NCT01971034	Completed. Poorly tolerated; No improvement in DCR.	Metformin +/− paclitaxel in gemcitabine refractory advanced PC
Metformin	mTOR-ATM/LKB1/AMPK	Phase II	NCT01210911	Completed. No improvement in OS.	Metformin +/− erlotinib + gemcitabine in advanced PC
Metformin	mTOR-ATM/LKB1/AMPK	Phase II	NCT01167738	Completed. No difference in PFS, DCR and OS	Metformin +/− capecitabine + cisplatin + epirubicin + gemcitabine in advanced PC
Metformin	mTOR-ATM/LKB1/AMPK	Phase II	UMIN000020681	Recruiting	Metformin +/− S−1 in resected PC
Metformin	mTOR-ATM/LKB1/AMPK	Phase II	NCT01666730	Completed. No results available	Metformin +/− mFOLFOX6 in metastatic PC
Metformin	mTOR-ATM/LKB1/AMPK	Phase III	NCT02201381	Completed. No results available.	Metfromin + atorvastatin + doxyciclin + mebendazole
Cixutumab	IGF-1R	Phase I/II	NCT00617708	Completed. No improvement in PFS and OS	Cixutumab +/− gemcitabine + erlotinib in metastatic PC
Ganitumab	IGF-1R	Phase III	NCT01231347	Completed. No improvement in PFS and OS	Ganitumab +/− gemcitabine in metastatic PC
Simvastatin	HMG-CoA reductase	Phase I	NCT03889795	Recruiting	Simvastatin + metformin + digoxin in advanced PC
Simvastatin	HMG-CoA reductase	Phase II	NCT00944463	Completed. No results available.	Simvastatin +/− gemcitabine in advanced PC

PFS = median Progression Free Survival; OS = Overall Survival; DCR = Disease Control Rate; TTP = Time to Progression.
